# Effects and therapeutic mechanism of Yinzhihuang on steatohepatitis in rats induced by a high‐fat, high‐cholesterol diet

**DOI:** 10.1111/1751-2980.12845

**Published:** 2020-02-27

**Authors:** Jing Zeng, Xiao Lin Liu, Feng Zhi Xin, Ze Hua Zhao, You Lin Shao, Rui Xu Yang, Qin Pan, Jian Gao Fan

**Affiliations:** ^1^ Department of Gastroenterology Xinhua Hospital, Shanghai Jiao Tong University School of Medicine Shanghai China; ^2^ Department of Gastroenterology The First Affiliated Hospital of Soochow University Suzhou Jiangsu Province China; ^3^ Shanghai Key Laboratory of Children's Digestion and Nutrition Shanghai China

**Keywords:** Chinese traditional medicine, high‐fat high‐cholesterol diet, network‐based regulatory model, non‐alcoholic fatty liver disease, therapeutic mechanism

## Abstract

**Objectives:**

We aimed to investigate the therapeutic mechanism of Yinzhihuang (YZH) liquid, a traditional Chinese medicine mainly composed of extracts of four components, on nonalcoholic steatohepatitis (NASH) induced by a high‐fat, high‐cholesterol diet (HFHCD) in rats.

**Methods:**

Altogether 30 Sprague‐Dawley rats were randomized into three groups: control, the model group (HFHCD + saline) and the treatment group (HFHCD + YZH). Liver histological features and serum biochemical parameters were assessed by the end of the 16th week. RNA sequencing and protein mass spectrometry detection were performed. The genes and proteins expressed differentially were subjected to KEGG pathway enrichment analysis and included in a network‐based regulatory model.

**Results:**

The weight, liver and fat indices and serum alanine transaminase, aspartate transaminase and total cholesterol levels of the HFHCD + YZH group were all significantly lower than those of the HFHCD + saline group. Moreover, their hepatic steatosis, ballooning and lobular inflammation were relieved, and 64 hepatic genes and 73 hepatic proteins were found to be reversed in their expression patterns after YZH treatment (*P* < 0.05). The network‐based regulatory model showed that these deregulated genes and proteins were mainly involved in oxidative phosphorylation, Toll‐like receptor, nucleotide‐binding oligomerization domain‐like receptor, peroxisome proliferator‐activated receptor signaling, nuclear factor‐kappa B tumor necrosis factor signaling pathways and fatty acid metabolism.

**Conclusion:**

YZH could alleviate NASH in HFHCD‐fed rats by inhibiting lipogenesis, accelerating lipid β‐oxidation, alleviating oxidative stress and relieving necroinflammation in the liver.

## 1 INTRODUCTION

The incidence of nonalcoholic fatty liver disease (NAFLD), which affects approximately one‐quarter of the global population, has been increasing over the past two decades.^1^ As an aggressive form of NAFLD, a definitive diagnosis of nonalcoholic steatohepatitis (NASH) is made based on histological evidence of hepatocellular injury and an accumulation of inflammatory cells in addition to hepatic steatosis.^2^ Simple steatosis usually runs a benign course in patients, while NASH is more likely to develop into progressive liver fibrosis and eventually cause liver‐related complications. This underscores the importance of developing interventions to prevent and treat NASH.^3^ Pharmacological therapy is required for patients with NASH or liver fibrosis as well as for those with morbid obesity or musculoskeletal disorders who are unable to exercise sufficiently.^4^ Despite its significant burden to the public healthcare system, no medication has yet been approved for the treatment of NASH by the Food and Drug Administration in the United States. Therefore, the need for appropriate treatment options and therapeutic targets is now quite urgent.^5^ Due to the complex pathophysiological process of NASH, effective therapy must target several key regulators of cellular and molecular events.

Long‐term effects of many monotherapies for systemic diseases have been found to be limited, and adverse events may present during the treatment course. The reasons may include the nature of the diseases, natural evolution of feedback loops and pathway redundancy. Traditional Chinese herbal medicines are rich in numerous bioactive substances with several biological properties, including anti‐inflammatory, anti‐infection and immunoregulatory properties.^6^ Traditional Chinese herbal medicine has been demonstrated to be effective for treating NAFLD.^7^ Yinzhihuang (YZH) oral solution, a traditional Chinese herbal medicine, is mainly composed of extracts of *Artemisia capillaris*, *Gardenia jasminoides* and *Scutellaria baicalensis*, with a small amount of *Lonicera japonica*.^8^ YZH has already been used to remove dampness to eliminate jaundice and detoxify the human body.

In clinical practice, YZH oral solution can be used in the treatment of neonatal jaundice and cholestasis. It can significantly reduce serum bilirubin levels, shorten the time for jaundice recovery, and ultimately achieve a high rate of therapeutic efficacy.^9^, ^10^ A clinical study in China reported that 12‐week therapy with YZH oral solution could significantly decrease serum levels of alanine transaminase (ALT), aspartate transaminase (AST), total cholesterol (TC), and triglyceride (TG) in patients with NAFLD, without causing any significant adverse reactions.^11^ YZH oral solution has also been reported to decrease serum aminotransferase and direct bilirubin levels in rats, together with a protective effect against intrahepatic cholestasis.^10^ In addition, it significantly decreased serum levels of ALT, AST, interleukin (IL)‐6, and tumor necrosis factor (TNF)‐α in mice with immunological liver injuries.^12^ Furthermore, our previous study showed that YZH oral solution could ameliorate liver histological features in rats with NASH induced by a methionine and choline‐deficient diet (MCD) or a high‐fat, high‐cholesterol diet (HFHCD).^13^, ^14^ Though these results suggest that YZH may be effective in the treatment of NAFLD or NASH, its therapeutic mechanism has not been clarified yet.

Complex multicomponent nature may make these traditional Chinese herbal medicines valuable resources due to their synergistic effects. In addition, multiple systems biology platforms have been shown to be the most powerful technologies available to uncover molecular mechanisms and connections between drugs and their multitargeted networks.^15^ Based on the rationale and technologies mentioned above, we used transcriptomics and proteomics platforms in the present study to analyze the functional mechanism of YZH oral solution in rats with HFHCD‐induced NASH and to investigate the relevant molecular targets and functional pathways from a network perspective.

## 2 MATERIALS AND METHODS

### 2.1 Preparation and analysis of YZH liquid

The YZH liquid was purchased from China Resources Sanjiu Medical & Pharmaceutical (Beijing, China). The identification and quantification of the marker compounds in the YZH liquid were performed according to the methods described in the Updated Pharmacopeia of the People's Republic of China.^16^ Briefly, 12 g of *Herba Artemisiae Capillariae*, 6.4 g of *Fructus Gardeniae* and 8 g of *L. japonica* were mixed and dissolved by adding 300 mL of double‐distilled water. The pH value was adjusted to 6.5 by adding 10% sodium hydroxide solution and the solution was filtered and reserved. *S. baicalensis* (40 g) was then dissolved in 300 mL of double‐distilled water and the pH value was adjusted to 6.5‐7.0 with a 10% sodium hydroxide solution. After filtration, the filtrate was mixed with the reserved solution. The compound was thoroughly mixed with 0.5 g of citric acid, 100 g of sucrose, 50 g of honey, 2 g of aspartame and 3 g of sodium benzoate. The mixture was refrigerated at 4–8 °C for 24 hours and the pH value was adjusted to neutral. An appropriate amount of double‐distilled water was added to this solution to adjust the total amount to 1000 mL. Finally, the solution was left to stand, filtered and sterilized to finally obtain the YZH liquid.

Marker compounds were analyzed using high‐performance liquid chromatography (Agilent 1260 Infinity; Agilent Technologies, Palo Alto, CA, USA) with a Venusil MP C18 column (250 mm × 4.6 mm, 5 μm; Agela Technologies, Wilmington, DE, USA), as described in a previous study.^17^ The reference substances for five marker compounds, including chlorogenic acid, geniposide, hyperoside, baicalin and luteolin, were purchased from the National Institutes for Food and Drug Control (Beijing, China). Measurements were all made at a flow rate of 1 mL/min and a detection wavelength of 327 nm. The column temperature was 35°C and the injection volume was 10 μL. The five characteristic peaks were identified by comparison with each reference substance (Figure S1).

### 2.2 Animal experiments

In total 30 male Sprague‐Dawley rats (6 weeks old) obtained from the Shanghai Experimental Animal Center of the Chinese Academy of Sciences (Shanghai, China) were kept (5 rats per cage) under controlled temperature (24 °C ± 2 °C) and humidity (50% ± 5%) with free access to food and water. The animal experiments were performed with reference to the National Research Council's Guide for the Care and Use of Laboratory Animals and with the approval of the Institutional Animal Care and Use Committee of Society for Human Resource Management (no. SHRM‐IACUC‐001).

**Figure 1:**
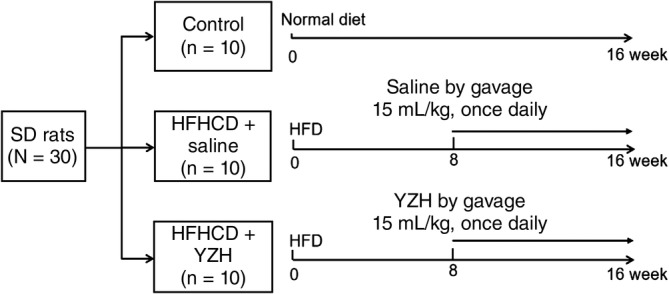
Flowchart of grouping of the control, high‐fat, high‐cholesterol diet (HFHCD) + saline, and HFHCD + Yinzhihuang (YZH) rats. The saline and YZH liquid were given to the rats by gavage once daily beginning at the end of the 8th week at doses of 15 mL/kg. HFHCD, high‐fat, high‐cholesterol diet; SD, Sprague‐Dawley rats, YZH, yinzhihuang

Following a week of acclimation, the rats were randomly divided into three groups using random number table. The control group was given a normal diet composing of 64.5% carbohydrates, 13.3% fat, and 22.2% protein. The model group (HFHCD + saline) was given a HFHCD (88% basic diet +10% lard +2% cholesterol, composing of 49.7% carbohydrates, 33.2% fat and 17.1% protein), and an addition of saline by gavage at a dose of 15 mL/kg once daily beginning at the end of the 8th week. The treatment group (HFHCD + YZH) was given a HFHCD and YZH liquid by gavage at a dose of 15 mL/kg once daily beginning at the end of the 8th week (Figure 1). All rats were sacrificed by cervical dislocation after an overnight fast at the end of the 16th week. Liver and epididymal fat were taken and weighed to calculate the liver index ([liver weight/body weight] × 100%) and fat index ([epididymal fat weight/body weight] × 100%), respectively. The liver tissues were fixed in 4% paragormaldehyde, frozen in O.C.T or snap‐frozen in liquid nitrogen and stored at ‐80 °C. The tissue blocks were cut into slices, and stained with hematoxylin and eosin for pathologic analysis, Oil Red O for detecting lipids and Masson for fibrosis. Serum biochemical indexes including ALT, AST, TC and TG, were measured using an automatic biochemical analyzer (Hitachi, Tokyo, Japan).

### 2.3 High‐throughput RNA sequencing and bioinformatics analysis

**Figure 2:**
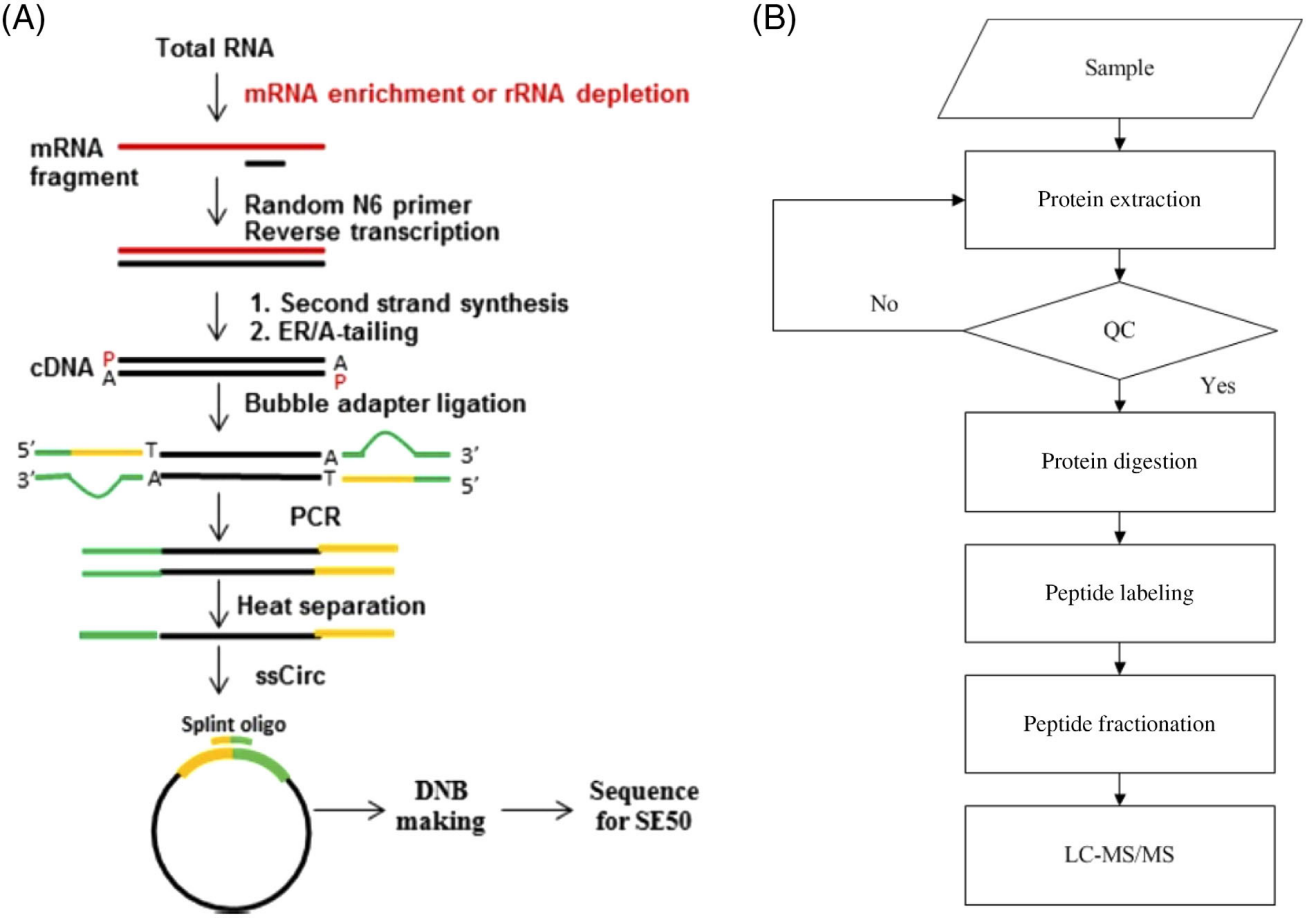
Main procedures of the RNA‐sequencing experiment and isobaric tags for relative and absolute quantification. A, the RNA sequencing experimental process; B, the main procedures of the iTRAQ quantitative proteomics experiment

Total RNA was extracted according to the manufacturer's protocol (TaKaRa, Shiga, Japan) from the livers of 12 rats (four rats per group). The RNA sequencing experimental process is shown in Figure 2A. The necessity of a resequencing step was determined by quality control of the primary sequencing data. After quality control, the raw reads were filtered into clean reads that were aligned to the reference sequences. The distribution of reads on the reference genes and the mapping ratio were calculated based on the alignment data. Then, gene expression analysis and deep analysis based on the gene expression data were included into the downstream analyses. Genes expressed differentially were screened according to the default criteria: a log2‐fold change ≥1.0 and a divergence probability ≥0.8. Furthermore, we performed a KEGG pathway enrichment analysis on the differentially expressed genes.

### 2.4 Isobaric tags for relative and absolute quantification (iTRAQ) proteomics and bioinformatics analysis

Protein was extracted from the remaining liver tissues of these 12 rats using a lysis buffer and two magnetic beads (diameter 5 mm). The main procedures of the iTRAQ quantitative proteomics experiment are shown in Figure 2B. Briefly, protein was quantified with a Bradford protein assay (Bio‐Rad, Foster City, CA, USA) and qualified by sodium dodecyl sulfate polyacrylamide gel electrophoresis (SDS‐PAGE). Trypsin was then used to digest the protein samples and iTRAQ reagents to label the peptides. The peptides were fractionated on a pump system Shimadzu LC‐20AB high‐performance liquid chromatography (HPLC) (Shimadzu, Kyoto, Japan). Each component in the mixture was separated, identified, and quantified by HPLC with LC‐20AD nano‐HPLC (Shimadzu). Finally, mass spectrometric detection was accomplished with TripleTOF 5600 (SCIEX, Framingham, MA, USA).

The exported mascot generic format (MGF) files were transformed based on the raw tandem mass spectrometry (MS)/MS data, and they were searched in the NCBI nr database (ftp://ftp.ncbi.nih.gov/blast/db). The peptides were labeled with isobaric tags and then quantitatively analyzed with the automated software IQuant (BGI‐Tech, Shenzhen, Guangdong Province, China). All proteins with a false discovery rate of less than 1% were subjected to downstream analysis. Furthermore, we performed a Kyoto Encyclopedia of Genes and Genomes (KEGG) pathway enrichment analysis on the differentially expressed proteins.

### 2.5 Establishment of a network‐based regulatory model

The generation of a network‐based regulatory model was based on the Cytoscape web application (Cytoscape Consortium, San Diego, CA, USA) with information obtained from several levels of functional analysis: the fold‐change analysis of genes or proteins, the protein–protein interaction analysis, the KEGG pathway enrichment analysis and the biological process enrichment analysis. The protein‐protein interaction analysis was accomplished by selecting genes and proteins expressed differentially via the widely used STRING biological database (https://string-db.org) and web resource. The default confidence cut‐off value was set as 400; solid lines were used between genes and proteins when interactions with scores were over 400, while dashed lines were used in interactions with scores lower than 400. Genes/proteins are represented with circular nodes, while KEGG pathways are indicated with rectangles. The pathways are colored in a gradient from yellow, indicating a larger *P* value, to blue, indicating a smaller *P* value. Genes/proteins are colored red (upregulated) and green (downregulated) in fold‐change analysis.

### 2.6 Statistical analysis

The data are presented as the mean ± standard error. One‐way ANOVA followed by a Newman‐Keuls post‐test was used to compare differences in continuous variables among groups with SPSS version 21.0 software (IBM, Armonk, NY, USA). Graphs were generated using GraphPad Prism Software version 6 (San Diego, CA, USA). Differences were considered statistically significant at *P* < 0.05.

## 3 RESULTS

### 3.1 Effects of YZH liquid on HFHCD‐induced NASH rats

**Figure 3:**
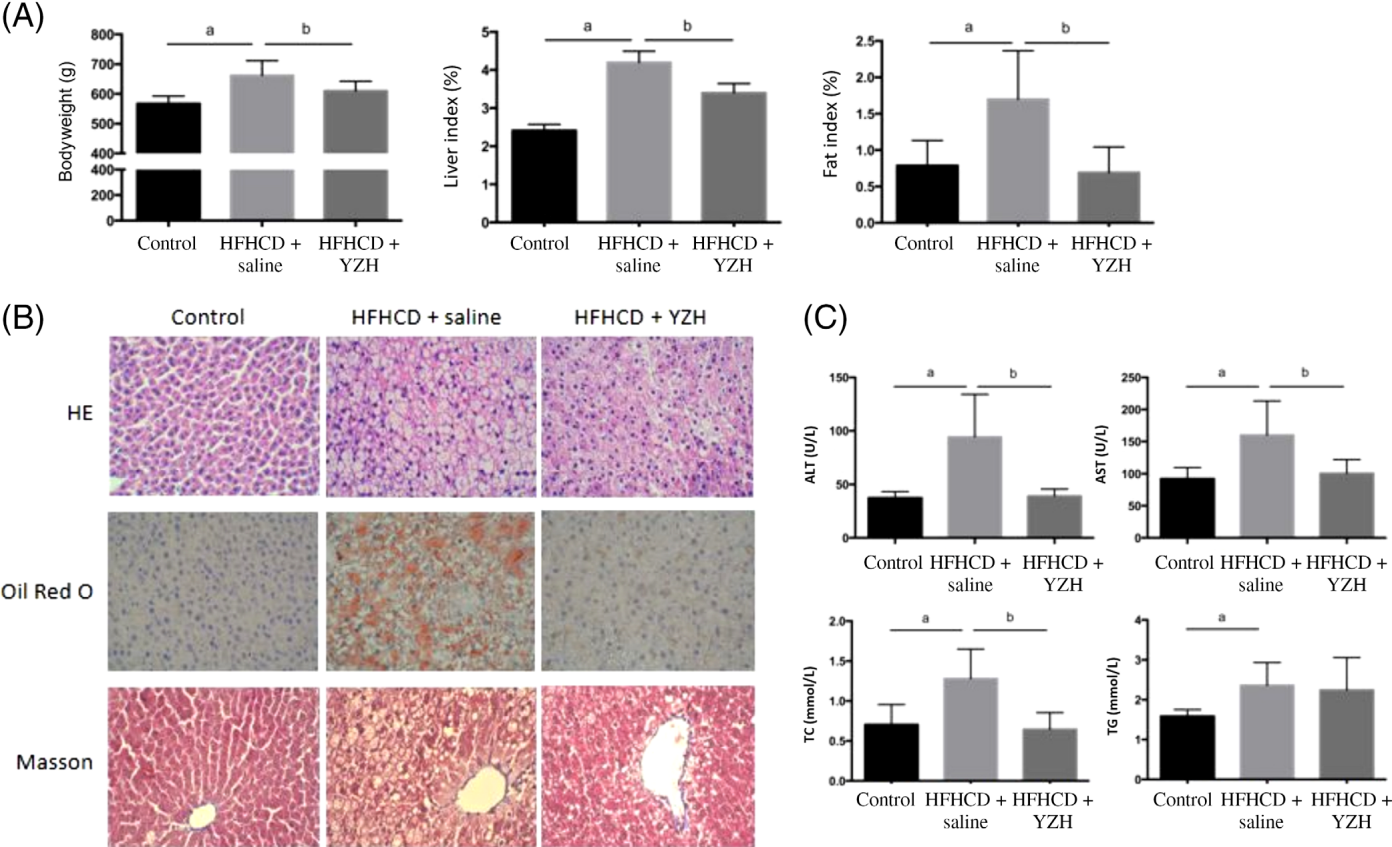
Effects of yinzhihuang (YZH) liquid on high‐fat, high‐cholesterol diet (HFHCD)‐induced nonalcoholic steatohepatitis (NASH). A, Effects of YZH liquid on body weight, liver index and fat index in NASH rats. B, Effects of YZH liquid on hepatic histological features of NASH rats as assessed by HE, Oil Red O, and Masson staining (×400). C, Effects of YZH liquid on serum alanine transaminase (ALT), aspartate transaminase (AST), total cholesterol (TC) and triglyceride (TG) levels. a, *P* < 0.05, HFHCD + saline vs control; b, *P* < 0.05, HFHCD + saline vs HFHCD + YZH

At the end of the 16th week, all rats in the HFHCD + saline group developed NASH with significant hepatocellular steatosis, ballooning and lobular inflammation. Compared with the control group, the HFHCD + saline group had a higher body weight, liver and fat index (*P* < 0.01), while the HFHCD + YZH group had a significantly lower body weight, liver index and fat index than the HFHCD + saline group (*P* < 0.01; Figure 3A). In addition, intake of YZH liquid improved hepatic histological features, including steatosis, ballooning and lobular inflammation (Figure 3B). Furthermore, serum ALT, AST and TC levels of the HFHCD + YZH group significantly decreased compared with those of the HFHCD + saline group (*P* < 0.05; Figure 3C), although they did not significantly differ compared with those of the control group.

### 3.2 Differentially expressed genes in liver tissues after YZH liquid administration

**Figure 4:**
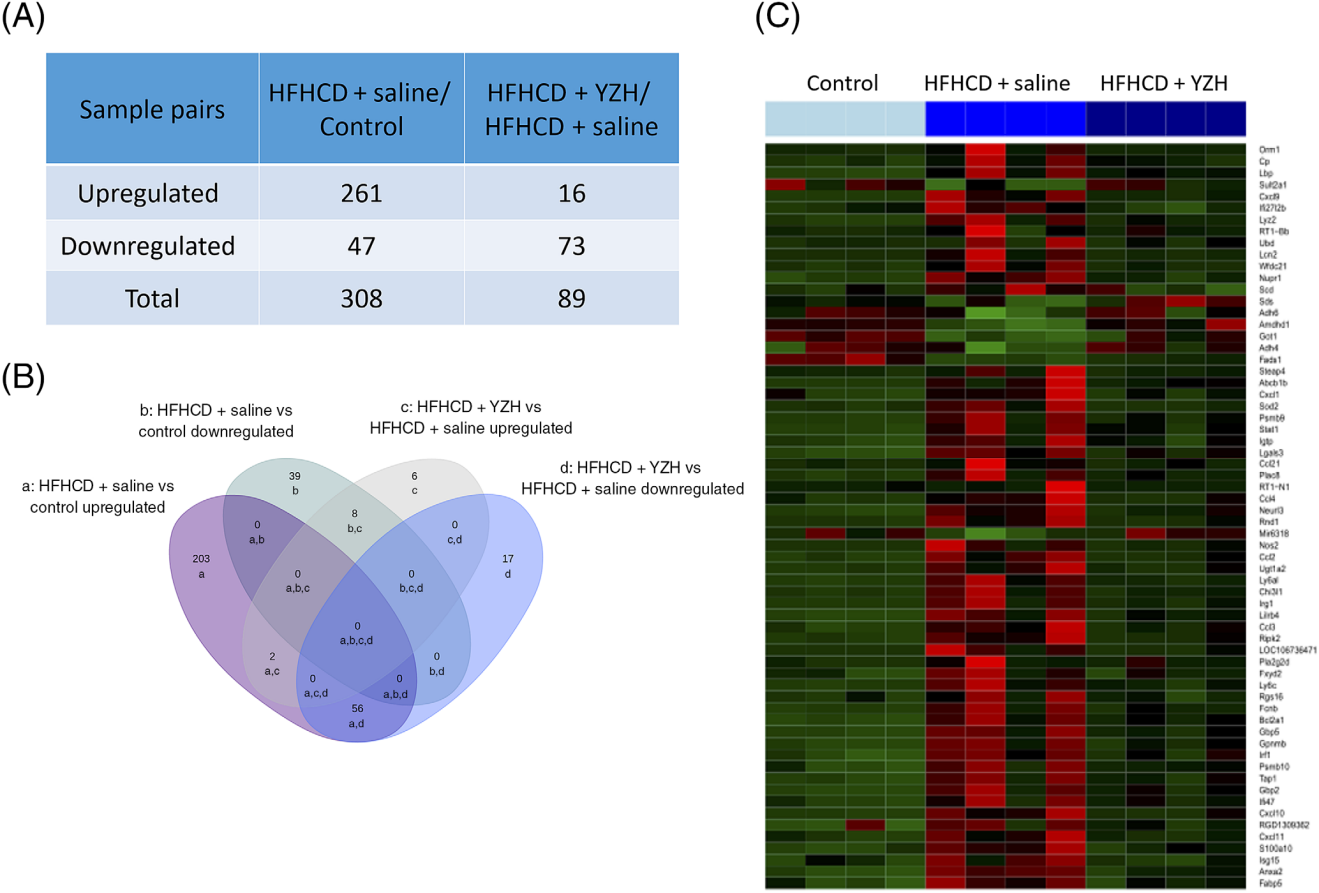
Differentially expressed hepatic genes after Yinzhihuang (YZH) liquid administration. A, Number of differentially expressed genes in the high‐fat, high‐cholesterol diet (HFHCD) + saline group compared with the control group or the HFHCD + YZH group. B, Venn diagram showing the overlap of the differentially expressed genes among the three groups: a, upregulated in HFHCD + saline vs control; b, downregulated when HFHCD + saline vs control; c, upregulated when HFHCD + YZH vs HFHCD + saline; d, downregulated when HFHCD + YZH vs HFHCD + saline. C, Heat map showing the hierarchical clustering of selected differentially expressed genes after YZH liquid administration. In the cluster analysis, the upregulated and downregulated genes are colored in red and green, respectively

Using the cut‐off criteria mentioned above, we identified 308 differentially expressed genes, including 261 upregulated genes and 47 downregulated genes, in the livers of HFHCD + saline rats compared with those of control rats. While compared with the HFHCD + saline rats, liver tissues of HFHCD + YZH rats exhibited 89 differentially expressed genes, with 16 upregulated and 73 downregulated genes (Figure 4A). Altogether 56 genes were found to be upregulated in the HFHCD + saline group while downregulated after treated with YZH liquid; however, eight genes were downregulated in HFHCD + saline but upregulated in HFHCD + YZH (Figure 4B). The expression patterns of these 64 hepatic genes were found to have been reversed after the administration of YZH liquid in the NASH rat models. A heat map of the gene expression analysis showing the hierarchical clustering of these differentially expressed genes after YZH administration in the different groups is shown in Figure 4C.

The KEGG pathway enrichment analysis included 64 selected genes which were expressed differentially. These genes were significantly enriched in 10 signaling pathways, including the chemokine signaling pathway, the Toll‐like receptor signaling pathway, the TNF signaling pathway, the nucleotide‐binding oligomerization domain (NOD)‐like receptor signaling pathway, biosynthesis of unsaturated fatty acids, the nuclear factor‐κB signaling pathway, fatty acid degradation, fatty acid metabolism, glycolysis and gluconeogenesis and bile secretion (Table 1).

**Table 1:** Pathway enrichment analysis for differentially expressed genes after oral administration of yinzhihuang liquid

	Pathways	Genes	N	*P* value
Immune system	Chemokine signaling pathway	*Ccl2, Ccl4, Ccl3, Stat1, Ccl21, Cxcl1, Cxcl11, Cxcl10, Cxcl9*	9	6.8E−08
Immune system	Toll‐like receptor signaling pathway	*Ccl4, Ccl3, Lbp, Stat1, Cxcl11, Cxcl10, Cxcl9*	7	2.1E−07
Signal transduction	TNF signaling pathway	*Ccl2, Cxcl1, Cxcl10, Ifi47*	4	1.5E−03
Immune system	NOD‐like receptor signaling pathway	*Ccl2, Cxcl1, Ripk2*	3	2.3E−03
Lipid metabolism	Biosynthesis of unsaturated fatty acids	*Fads1, Scd1*	2	7.6E−03
Signal transduction	NF‐kB signaling pathway	*Ccl4, Lbp, Ccl21*	3	9.0E−03
Lipid metabolism	Fatty acid degradation	*Adh6, Adh4*	2	1.9E−02
Lipid metabolism	Fatty acid metabolism	*Fads1, Scd1*	2	2.5E−02
Carbohydrate metabolism	Glycolysis and gluconeogenesis	*Adh6, Adh4*	2	4.0E−02
Digestive system	Bile secretion	*Fxyd2, Abcb1b*	2	4.2E−02

Abbreviations: NF‐κB, nuclear factor‐kappa B; NOD, nucleotide‐binding oligomerization domain; TNF, tumor necrosis factor.

### 3.3 Differentially expressed hepatic proteins after YZH liquid administration

**Figure 5:**
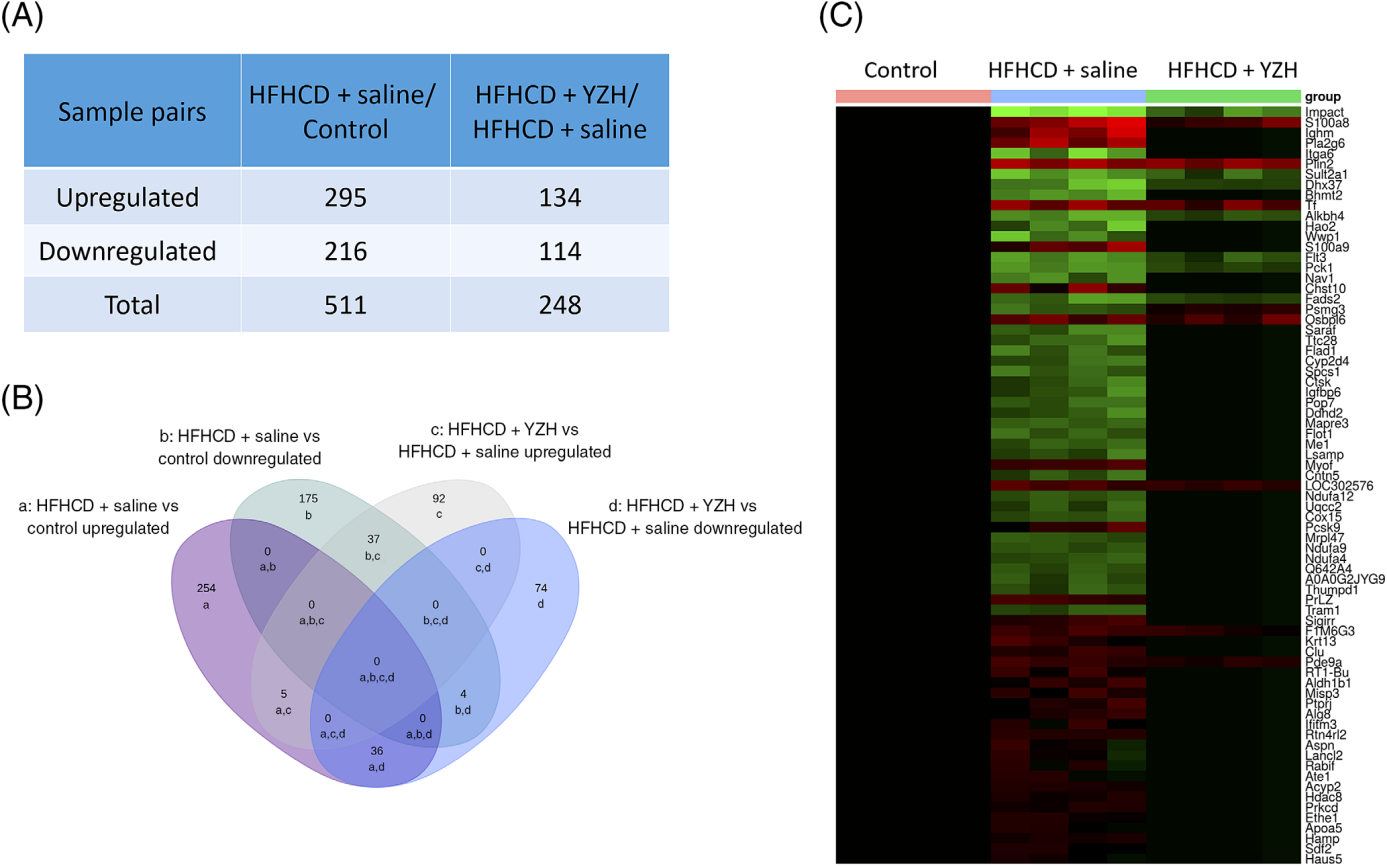
Differentially expressed hepatic proteins after Yinzhihuang (YZH) liquid administration. A, Number of differentially expressed proteins in the high‐fat, high‐cholesterol diet (HFHCD) + saline group vs the control group and in the HFHCD + YZH group vs the HFHCD + saline group. B, Venn diagram showing the overlap of the differentially expressed proteins after YZH liquid therapy. a, upregulated when HFHCD + saline vs control; b, downregulated when HFHCD + saline vs control; c, upregulated when HFHCD + YZH vs HFHCD + saline; d, downregulated when HFHCD + YZH vs HFHCD + saline. C, Heat map showing the hierarchical clustering of selected differentially expressed proteins after YZH liquid therapy. The expression levels of the selected proteins in the control group were defined as the baseline values

Altogether 24 692 peptides and 4967 proteins were identified in this proteomics analysis with a 1% false discovery rate. The analysis revealed that 295 proteins were upregulated and 216 were downregulated in the HFHCD + saline group compared with the control group, while 134 proteins were upregulated and 114 downregulated in the HFHCD + YZH group compared with the HFHCD + saline group (Figure 5A). Among these deregulated proteins, 36 upregulated proteins in the HFHCD + saline group were downregulated after the YZH administration. Additionally, 37 proteins that had downregulated in the HFHCD + saline group were upregulated in the HFHCD + YZH group (Figure 5B). The expression levels of these 73 differentially expressed proteins are displayed in detail in a heat map (Figure 5C).

Based on the results of the KEGG pathway enrichment analysis, the 73 selected differentially expressed proteins were classified into four main signaling pathways, including oxidative phosphorylation, the peroxisome proliferator‐activated receptor (PPAR) signaling pathway, alpha‐linolenic acid metabolism, and metabolic pathways (Table 2).

**Table 2:** Pathway enrichment analysis for differentially expressed proteins after Yinzhihuang liquid administration

	Pathways	Genes	N	*P* value
Energy metabolism	Oxidative phosphorylation	*Ndufa4, Ndufa12, Ndufa9, Cox15*	4	4.3E−04
Endocrine system	PPAR signaling pathway	*Apoa5, Pck1, Fads2*	3	1.1E−03
Lipid metabolism	alpha‐linolenic acid metabolism	*Pla2g6, Fads2*	2	1.8E−03
Metabolic pathways	Metabolic pathways	*Pla2g6, Ndufa4, Ndufa12, Ndufa9, Cox15, Hao2, Pck1, Bhmt2*	8	1.1E−02

Abbreviation: PPAR, peroxisome proliferator‐activated receptor.

### 3.4 Network‐based regulatory model of YZH liquid on NASH

**Figure 6:**
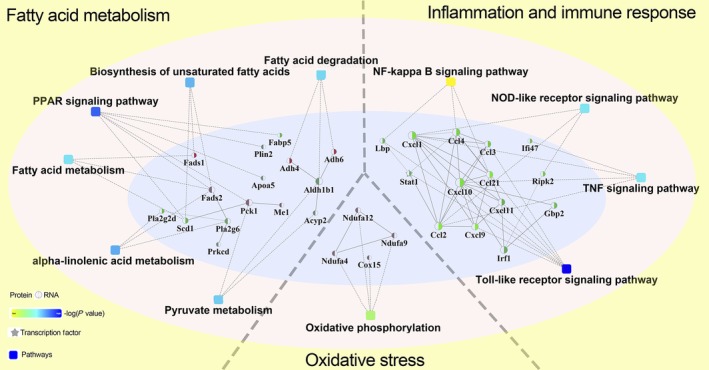
Network‐based regulatory model of Yinzhihuang (YZH) liquid for nonalcoholic steatohepatitis. The 64 differentially expressed genes and 73 differentially expressed proteins after YZH liquid administration were selected to establish a gene–protein interaction network. The circular nodes indicated genes or proteins, while the rectangles indicated KEGG pathways. The pathways are colored with a gradient from yellow to blue, with yellow color indicates a larger *P* value, while blue color indicates a smaller *P* value. In the case of fold‐change analysis, the genes/proteins are colored red (upregulated) and green (downregulated), respectively. Abbreviations: NOD, nucleotide‐binding oligomerization domain; PPAR, peroxisome proliferator‐activated receptor; TNF, tumor necrosis factor

The 64 differentially expressed genes and 73 differentially expressed proteins after YZH administration were selected to establish a gene–protein interaction network (Figure 6). The results indicate that there are 33 genes or proteins involved in the proposed regulatory model. The *Fads1*, *Fads2*, *Pck1*, *Me1*, *Adh4* and *Adh6* were upregulated, and *Scd1*, phospholipase A2 (*Pla*) family members (including *Pla2g2d* and *Pla2g6*), *Prkcd*, *Fabp5*, *Plin2*, *Apoa5*, *Aldh1b1*, and *Acyp2* downregulated and were involved in the PPAR signaling pathway, biosynthesis of unsaturated fatty acids, fatty acid degradation, fatty acid metabolism, alpha‐linolenic acid metabolism and pyruvate metabolism, which were mostly associated with fatty acid metabolism. Meanwhile, the upregulated *Ndufa4*, *Ndufa9*, *Ndufa12* and *Cox15* were involved in oxidative phosphorylation, which is a pathway regulating oxidative stress. Genes including *Lbp*, *Stat1*, *Ifi47*, *Ripk2*, *Gbp2*, *Irf1*, *Ccl2*, *Ccl3*, *Ccl4*, *Ccl21*, *Cxcl1*, *Cxcl9*, *Cxcl10* and *Cxcl11* were all downregulated. They were mostly involved in inflammation and immune response pathways, including the NF‐κB, Toll‐like receptor, NOD‐like receptor, and TNF signaling pathways. Overall, the network‐based regulatory model indicated that YZH liquid could alleviate NASH in HFHCD‐fed rat models by regulating fatty acid metabolism, oxidative stress, inflammation and immune responses.

## 4 DISCUSSION

The mechanism remains unknown although our previous studies have demonstrated the effectiveness of YZH liquid on MCD‐ and HFHCD‐induced NASH in animal models. In the current study HFHCD‐induced NASH rat model was used, which is more characteristic of the natural course of human NASH compared with MCD‐induced model. We found that YZH liquid not only reduced the body weight, liver and fat index of the NASH rats but also improved hepatic histological features and decreased serum transaminase levels. Importantly, for the first time, we combined RNA sequencing with protein profiling to explore the mechanism by which YZH liquid alleviated NASH and constructed a network‐based regulatory model. Based on this model, YZH liquid might inhibit lipogenesis and accelerate lipid β‐oxidation by regulating fatty acid metabolism and the PPAR signaling pathway, alleviate oxidative stress by upregulating oxidative phosphorylation and relieve intrahepatic inflammation through downregulating Toll‐like receptor, NOD‐like receptor, NF‐κB and TNF signaling pathways.

Resulting from the imbalance between lipid deposition and removal, hepatic fat accumulation can be regulated by nuclear receptors and cytoplasmic transcription factors, including PPAR family members.^18^ The three PPAR isotypes regulate fatty acid uptake, lipogenesis, fatty acid oxidation, oxidative stress and inflammation of the liver.^19^ Although it does not influence the expression level of PPARs themselves, YZH liquid regulates important genes and proteins involved in PPAR signaling pathways. As a key regulatory enzyme at the last stage of hepatic de novo lipogenesis, enhanced hepatic SCD1 activity promotes the accumulation of hepatic lipids, consequently leading to the progression of fatty liver.^20^ FABP5 contributes to the development of diet‐induced obesity.^21^ PLIN2 is involved in the elevation of circulating free fatty acid concentrations.^22^ APOA5 is a liver‐specific protein that promotes hepatic TG storage and contributes to the pathogenesis of NAFLD.^23^ In our study, YZH liquid could suppress the expression of these lipogenic genes. In addition, YZH could also promote the activity of PCK1 and FADS2. Increased hepatic PCK1 protein concentrations have been reported to induce pyruvate cycling and accelerate lipid β‐oxidation.^24^ *Fads* expression has been found to be negatively associated with hepatic fat content, and FADS can suppress the accumulation of hepatic lipids.^25^ In addition, YZH liquid decreased the expression levels of *Pla2* family members, which are upstream checkpoints regulating fatty acid uptake that are upregulated in patients with NASH.^26^, ^27^ The knockout of *Pla2* has been proven to achieve a 56.5% decrease in fatty acid influx in hepatocytes.^26^ The administration of YZH liquid also increases the expression of alcohol dehydrogenase, the impairment of which has been noticed in the liver tissue of NASH patients.^28^ Overall, YZH liquid reduces hepatic lipid accumulation in HFHCD‐fed NASH rats by limiting lipogenesis and accelerating β‐oxidation.

The accumulation of TG in hepatocytes is caused by increased lipogenesis with decreased fatty acid β‐oxidation, which, combined with increased levels of reactive oxygen species mainly generated in mitochondria, contributes to the development of NASH.^29^ Our experiment demonstrated a significant decrease in the expression of the mitochondrial respiratory chain complex subunits in NASH, such as *Ndufa4*, *Ndufa9*, *Ndufa12* and *Cox15*. The impairment of mitochondrial oxidative phosphorylation can decrease adenosine triphosphate production and inhibit lipid β‐oxidation, which leads to hepatocellular steatosis, thus initiate oxidative stress and subsequent development of necroinflammation.^30^, ^31^ After YZH liquid administration, the number of MRC complexes in NASH rat livers increased significantly, indicating the recovery of mitochondrial function. This recovery could promote lipid β‐oxidation and reduce oxidative stress.

Hepatocyte injury is the key feature that differentiates NASH from isolated steatosis.^32^ Injured hepatocytes release factors promoting the accumulation of immune cells that produce hepatotoxic substances and induce further injury and inflammation.^33^, ^34^ In our study, the genes involved in the Toll‐like receptor signaling pathway, the NF‐κB signaling pathway, the NOD‐like receptor signaling pathway, and TNF signaling pathway were significantly increased in the NASH rat models. These genes, including *Lbp*, *Stat1*, *Ripk2*, *Irf1*, *Gbp2* and several chemokines, were suppressed after YZH liquid therapy. Chemokines are chemoattractants for leukocyte trafficking, growth and activation at sites of injury and inflammation, and in the livers of patients with NASH, the expression of chemokines and their receptors was increased based on accumulated data.^35^ Studies have demonstrated the successful pharmacological inhibition of hepatic monocyte/macrophage infiltration and amelioration of steatosis and inflammation development by the inhibition of *Ccl2* or *Cxcl10*, which links hepatocyte lipotoxicity to macrophage‐associated liver inflammation in NASH as a potent chemotactic ligand.^36^, ^37^ There is evidence that the endotoxemia marker *Lbp* is increased in patients with NASH and this correlates with lobular inflammation,^38^ and the knockout of *Lbp* in mice can decrease indices of liver damage determined by liver histology and serum transaminases.^39^ *Stat1* serves as a mediator in interferon γ and chemokine expression induced by lipopolysaccharide in macrophages, and the inhibition of Stat1 can inhibit the release of chemokines and suppress the amplification cascade of inflammatory signals.^40^ Therefore, YZH liquid may also alleviate the inflammatory cascade reaction in NASH rat livers through the downregulation of chemokines and other inflammation‐related factors.

There were some limitations to our study. First, TG levels were measured only in the serum, while its accumulation in hepatocytes is actually the main component of hepatic steatosis. Second, the therapeutic mechanism of YZH liquid was evaluated in NASH rat models. The efficacy and functional mechanism of this herbal medicine need further evaluation in clinical studies. Third, the specific regulatory relationships between genes and proteins in this network‐based regulatory model have not been fully elucidated in our study. Therefore, the verification of RNA sequencing in several selected pathways is needed and functional experiments need to be specifically designed to support our claims.

## 5 CONCLUSION

In conclusion, YZH liquid was effective in reducing body weight and serum aminotransferase levels and alleviating pathological liver injury in HFHCD‐fed NASH models. The network‐based regulatory model of YZH liquid on NASH shows that YZH liquid can reduce lipid deposition in the liver by inhibiting lipogenesis and accelerating lipid β‐oxidation, and can alleviate oxidative stress by upregulating the expression of mitochondrial respiratory chain complex. Finally, YZH liquid relieves intrahepatic inflammation by downregulating chemokines and other inflammation‐related factors. These results provide a theoretical basis for undergoing a randomized clinical trial of YZH liquid on patients with NASH in the near future.

## CONFLICT OF INTERESTS

The authors declare that they have no competing interests.

## Supporting information


**Figure S1.** Marker compounds in yinzhihuang liquid measured by high‐performance liquid chromatography at 327 nm. A, Negative control. B, Mixture control. C, yinzhihuang liquid sample. 1, chlorogenic acid; 2, geniposide; 3, hyperoside; 4, baicalin; 5, luteolin.
