# Supplementary figures and images for: Effects and therapeutic mechanism of Yinzhihuang on steatohepatitis in rats induced by a high‐fat, high‐cholesterol diet
Source: J Dig Dis. 2020 Feb 27;21(3):179–88. doi: 10.1111/1751-2980.12845 (PMC7187410; doi:10.1111/1751-2980.12845)

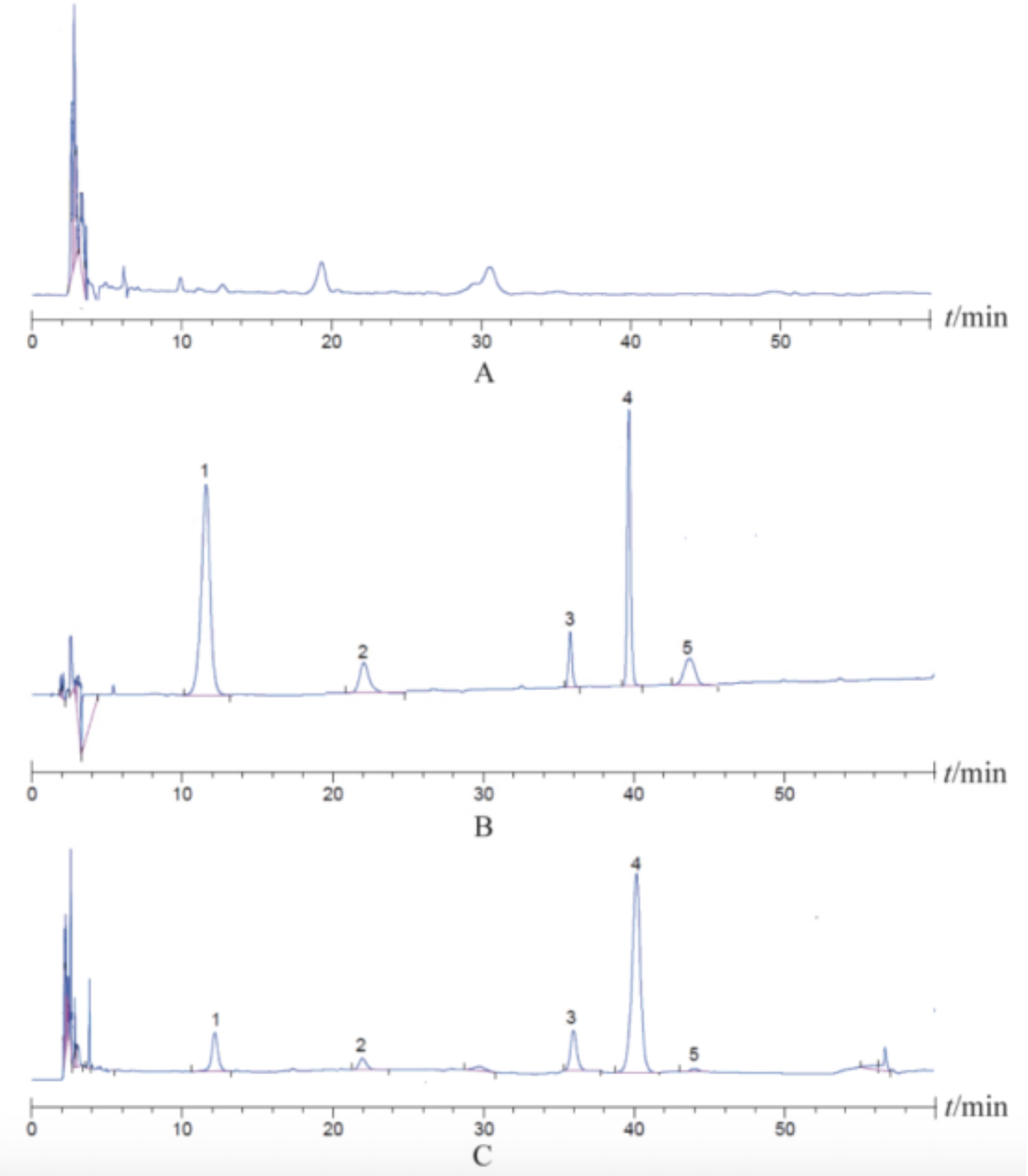

Supplement: Supplementary file 1 — Figure S1. Marker compounds in yinzhihuang liquid measured by high‐performance liquid chromatography at 327 nm. A, Negative control. B, Mixture control. C, yinzhihuang liquid sample. 1, chlorogenic acid; 2, geniposide; 3, hyperoside; 4, baicalin; 5, luteolin. [file CDD-21-179-s001.jpg]
